# Literacy-adapted, electronic family history assessment for genetics referral in primary care: patient user insights from qualitative interviews

**DOI:** 10.1186/s13053-022-00231-3

**Published:** 2022-06-10

**Authors:** Kathleen F. Mittendorf, Hannah S. Lewis, Devan M. Duenas, Donna J. Eubanks, Marian J. Gilmore, Katrina A. B. Goddard, Galen Joseph, Tia L. Kauffman, Stephanie A. Kraft, Nangel M. Lindberg, Ana A. Reyes, Elizabeth Shuster, Sapna Syngal, Chinedu Ukaegbu, Jamilyn M. Zepp, Benjamin S. Wilfond, Kathryn M. Porter

**Affiliations:** 1 Vanderbilt-Ingram Cancer Center, Vanderbilt University Medical Center, 2525 West End Avenue, Nashville, TN 37203 USA; 2 Treuman Katz Center for Pediatric Bioethics, Seattle Children’s Research Institute, 1900 9th Ave, Seattle, WA 98101 USA; 3 Center for Health Research, Kaiser Permanente Northwest, 3800 N. Interstate Ave, Portland, OR 97227 USA; 4 Department of Translational and Applied Genomics, Center for Health Research, Kaiser Permanente Northwest, 3800 N. Interstate Ave, Portland, OR 97227 USA; 5 Division of Cancer Control and Population Sciences, National Cancer Institute, 9609 Medical Center Drive, Bethesda, MD 20892 USA; 6 Department of Humanities and Social Sciences, University of California San Francisco, 490 Illinois Street, 7th Floor, San Francisco, CA 94143 USA; 7 Department of Pediatrics, Division of Bioethics and Palliative Care, University of Washington, 1959 NE. Pacific St, Seattle, WA 98195 USA; 8 Dana-Farber Cancer Institute, 450 Brookline Ave, Boston, MA 02215 USA; 9 Harvard Medical School, 25 Shattuck St, Boston, MA 02115 USA; 10 Brigham and Women’s Hospital, 75 Francis St, Boston, MA 02115 USA

**Keywords:** Underserved, Hereditary cancer risk assessment, Digital health, Genetics

## Abstract

**Background:**

Risk assessment for hereditary cancer syndromes is recommended in primary care, but family history is rarely collected in enough detail to facilitate risk assessment and referral – a roadblock that disproportionately impacts individuals with healthcare access barriers. We sought to qualitatively assess a literacy-adapted, electronic patient-facing family history tool developed for use in diverse, underserved patient populations recruited in the Cancer Health Assessments Reaching Many (CHARM) Study.

**Methods:**

Interview participants were recruited from a subpopulation of CHARM participants who experienced barriers to tool use in terms of spending a longer time to complete the tool, having incomplete attempts, and/or providing inaccurate family history in comparison to a genetic counselor-collected standard. We conducted semi-structured interviews with participants about barriers and facilitators to tool use and overall tool acceptability; interviews were recorded and professionally transcribed. Transcripts were coded based on a codebook developed using inductive techniques, and coded excerpts were reviewed to identify overarching themes related to barriers and facilitators to family history self-assessment and acceptability of the study tool.

**Results:**

Interviewees endorsed the tool as easy to navigate and understand. However, they described barriers related to family history information, literacy and language, and certain tool functions. Participants offered concrete, easy-to-implement solutions to each barrier. Despite experience barriers to use of the tool, most participants indicated that electronic family history self-assessment was acceptable or preferable in comparison to clinician-collected family history.

**Conclusions:**

Even for participants who experienced barriers to tool use, family history self-assessment was considered an acceptable alternative to clinician-collected family history. Barriers experienced could be overcome with minor adaptations to the current family history tool.

**Trial registration:**

This study is a sub-study of the Cancer Health Assessments Reaching Many (CHARM) trial, ClinicalTrials.gov, NCT03426878. Registered 8 February 2018.

**Supplementary Information:**

The online version contains supplementary material available at 10.1186/s13053-022-00231-3.

## Background

Hereditary cancer syndromes result from heritable pathogenic genetic variants that increase lifetime cancer risk, cancer at younger ages, and disease aggressiveness [1–5]. These syndromes affect at least 1 in 200 individuals [2–4, 6–8]. Since 2013, the United States Preventive Services Task Force (USPSTF) has recommended family history risk assessment for hereditary breast and ovarian cancer syndrome (HBOC) in primary care [9–12]. This recommendation has not been widely implemented, and medically underserved populations are less likely to have risk assessment, genetics referral, and genetic testing [13–20]. Even when clinicians collect family history information, it often lacks detail or clinicians do not make indicated referrals [18–21].

Patient-facing electronic family history tools are a promising approach to close these care gaps [22–25]. However, reviews of existing tools have identified critical areas to address to ensure equitable implementation in underserved populations [24–28]. For example, significant differences have been observed in tool completion by race/ethnicity and insurance status [29, 30].

The Cancer Health Assessments Reaching Many (CHARM) study designed a multimodal intervention to address care disparities in hereditary cancer services [31] and tested the intervention in a population enriched for individuals from medically underserved populations. As one intervention component, we designed a literacy-adapted, electronic, patient-facing family history self-assessment and automated risk assessment application [32]. Most participants were able to complete the self-assessment in a timely fashion and accurately report their family history, but some had incomplete attempts, spent considerably more time using the tool, and/or provided inaccurate family history [32].

To ensure equitable implementation of family history self-assessment tools, it is critical to understand barriers to their use [24, 26–28]. Accordingly, we conducted semi-structured qualitative interviews with individuals who experienced barriers to using the study tool. Here, we describe participant-identified barriers and facilitators, participant-identified solutions to experienced barriers, and participant-described acceptability of family history self-assessment and automated risk assessment as an alternative to family history collection and risk assessment by a clinician.

## Methods

### Setting and study design

The CHARM study is part of the Clinical Sequencing Evidence-Generating Research (CSER) Consortium [33]. The CHARM setting and study design have been previously described elsewhere [31]. Participants ages 18–49 were recruited from Kaiser Permanente Northwest (KPNW) in the Portland, Oregon metropolitan area, and Denver Health (DH), in Denver County, Colorado. Recruitment efforts were enriched for medically underserved populations at both sites based on previously defined methods [31]. The study defined enrolled participants with one or more of the following barriers to healthcare access as belonging to medically underserved populations: (1) Hispanic ethnicity or a race other than White; (2) residing in a Health Resources and Services Administration–defined medically underserved census tract; (3) Spanish language preference for the risk assessment or any subsequent study survey; (4) educational attainment less than high school graduate; (5) income < 200% of the Federal Poverty Level; (6) use of Medicaid insurance or being uninsured; (7) sexual orientation other than heterosexual; (8) gender identity other than cisgender female/male [34]. The KPNW Institutional Review Board (IRB) approved this study and all collaborating IRBs ceded to KPNW or approved the study separately.

Following initial recruitment, patients were asked to complete the family history assessment tool, which has been previously described [31, 32]. This patient-facing web tool was designed using a mobile-first approach and literacy-adapted from clinician-facing versions of two guideline-recommended validated hereditary cancer risk assessments: the B-RST™ 3.0 for HBOC risk [11, 35], and PREMM®_5_ for Lynch syndrome (LS) risk [8, 36–38]. We developed a novel algorithm to evaluate limited family structure/knowledge in accordance with National Comprehensive Cancer Network guidelines [7, 8, 32]. Responses to preliminary questions about personal and family cancer history determine whether patients are initially pathed to the B-RST™ 3.0 and PREMM®_5_ modules or are sent directly to the limited family history module. Patients who are exposed to B-RST™ 3.0 and PREMM®_5_ but fail to screen at risk by either algorithm are then exposed to the limited family history module; both algorithms require completions of all questions to obtain necessary variables. Individuals in the CHARM study who screened at increased risk or as having limited family history/structure were offered genetic testing. Family history was re-assessed by genetic counselors during result disclosure.

### Recruitment to participant interviews

Interviewees were recruited from both sites by phone or email between February and July 2020. Non-respondents were recontacted by up to two follow-up phone calls. Participants who took the risk assessment in Spanish were excluded because the interviewer (KFM) was monolingual.

KPNW participants who had at least one incomplete attempt of the risk assessment (“incompletion cohort”) were recruited within 3 months of last incomplete attempt. For DH participants, the time since last incomplete attempt was expanded to six months to broaden the recruitment pool, as the number of individuals identified in the three-month window was small. We recruited equal numbers of individuals with incomplete attempts who failed to ever complete the tool and those who later successfully completed it.

Participants with inconsistent family history reports between the tool and genetic counselor-collected family history (“accuracy cohort”) were recruited within six months of tool completion based on 1) discrepancy between their risk result from the tool recorded in the automated tracking system and their risk result derived from genetic counselor-collected family history recorded in an internal database, or 2) genetic counselor referral to this subproject.

Participants within the upper quartile of time-to-completion (“time cohort”) based on an interim analysis of completion time in the automated tracking system were recruited within 3 months of tool completion, unless they also met criteria for the accuracy cohort (6-month window). We excluded participants who opted to receive study team assistance to complete the tool when deriving this cohort, as this process could take over 30 minutes.

### Semi-structured interviews

We developed a semi-structured interview guide to evaluate participants’ experiences with the tool based on study questions and literature review. This guide was iteratively refined based on pilot testing and input from experts in qualitative analysis, health literacy, and tool development, and from two patient advisors [32]. Most questions in the interview guide were shared among the three cohorts, except questions addressing incompletion, time spent on the tool, and accuracy of family history report. Participants could belong to multiple cohorts, and interviewers included all applicable questions for an individual. Additional file 1 contains key questions about barriers, facilitators, solutions, and acceptability (see Additional file 1).

Interviews were conducted in English by a trained interviewer (KFM) via phone calls through secure Microsoft® Teams software, recorded with consent to a secure server, and professionally transcribed. Transcripts were redacted for potentially identifying information, then uploaded to cloud-based Dedoose software (https://www.dedoose.com) for coding and analysis.

### Analysis

An initial codebook (Additional file 2) was developed using inductive techniques [39] based on interview guide topic areas; codes were added based on high-level review of transcripts and analysis team consensus. Test coding was completed by a subset of the analysis team, who made codebook modifications until they consistently reached consensus. The larger analysis team provided final input prior to coding.

Dual coding and consensus review were completed for the first four transcripts in each cohort by two coders (KFM, HSL) and a tie breaker (KMP). Thereafter, a single coder (KFM or HSL) completed coding with dual coding occurring every 3–5 interviews to ensure consensus was maintained; therefore, over one-third of interviews in each cohort were dual-coded. When requested by the single coder, the second coder reviewed portions of select interviews. Tiebreaking (KMP) occurred when consensus was not reached.

Code excerpts were reviewed to identify themes related to barriers and facilitators of tool use and acceptability compared to clinician-collected family history.

## Results

### Interview participant characteristics

A total of 102 individuals were contacted for recruitment, with fifty-four individuals responding and consenting to interview. The incompletion cohort consisted of 20 individuals: ten who had not completed the tool at time of interview, and ten who had completed the tool prior to interview following their incomplete attempt(s). Of those who had not completed the assessment at time of interview, two individuals later completed it. Thirty-three interviewed participants met time and/or accuracy cohort criteria: 12 only for time, 14 only for accuracy, and seven met both. Interviewee characteristics are presented in Table 1 (per-cohort characteristics in Additional file 3). Similar barriers and facilitators were described in each cohort, so results are presented collectively, with differences between cohorts indicated where appropriate. Only five participants from DH were successfully recruited (Table 1), so site-specific analyses were not performed.

**Table 1:** Interviewee characteristics

Characteristic^a^	N (%)
Sex assigned at birth	
Male	7 (13)
Female	47 (87)
Gender Identity	
Male	7 (13)
Female	42 (78)
No response	5 (9)
Race or ethnicity	
Non-Hispanic White	16 (30)
Black/African American	5 (9)
Asian	5 (9)
Hispanic/Latino	15 (28)
AI/NA/AN	2 (4)
Middle Eastern/North African/Mediterranean	2 (4)
Native Hawaiian/Pacific Islander	1 (2)
Multi-racial/-ethnic	4 (7)
No response	4 (7)
Education	
Some high school	1 (2)
High school/equivalent	1 (2)
Some post high school	3 (6)
Associate/equivalent^b^	12 (22)
Bachelors	16 (30)
Masters	12 (22)
Doctorate/professional	5 (9)
No response	4 (7)
Annual household income	
Less than $20,000	1 (2)
$20–39,999	8 (15)
$40–59,999	7 (13)
$60–79,999	12 (22)
$80–99,999	7 (13)
$100–139,999	10 (19)
$140,000 +	5 (9)
No response	4 (7)
Insurance type	
Private, employer	39 (72)
Private, purchased	5 (9)
Government, Medicaid	5 (9)
Uninsured	1 (2)
No response	4 (7)
Recruitment site	
Kaiser Permanente Northwest	49 (91)
Denver Health	5 (9)
Cohort	
Incompletion	20 (37)
Time	13 (24)
Accuracy	14 (26)
Time + Accuracy	7 (13)
Mean age (range)	38.1 (23–50)

^a^ Source: Study tracking system data based on participant input (sex assigned at birth, age), study surveys or interviews (all other demographics), and/or analytic comparisons of scores derived from participant input on the study tool (study tracking system database) to genetic counselor risk scores record in a separate database

^b^ Associate (2-year) college degree, or completed occupational, technical, or vocational program and received degree or certificate

### Participant experiences

Participants described several barriers and facilitators to tool use, with most shared across all cohorts. The two most common themes were needing to acquire family history data from other family members and challenges accessing family history. Participants also discussed language accessibility for those who spoke English as a second language, literacy accessibility, and tool design elements.

#### Gathering family history

The most frequent reason for stopping the assessment, spending more time on the assessment, and/or misreporting information on the assessment compared to genetic counselor-collected information was needing to ask more knowledgeable relatives for family history information. An interviewee explained:

“I think I had to text my mom to ask her if her sister, my aunt, had that. I was waiting for a text back…So it was just a timeframe of waiting for information.” (Time; Interviewee 29).

Many participants who identified this barrier suggested or endorsed a solution in which recruitment materials and the tool provide a prominent notification encouraging family history gathering in advance and indicating the level of detail needed (Table 2).

**Table 2:** Barriers to using the risk assessment tool and suggested solutions

Barrier type	Suggested Solution(s)	Example Quote(s)
Access to family history	• Add “I don’t know” options • Move limited family history module to beginning and use it to drive skip logic	“I think that if you were to do that, like have that question at the start and then have that sort of be a gate for having the questions about say your father’s family history, that might have been helpful.” (Incompletion, Interviewee 20)
Acquisition of family history	• Add notification to acquire family history in advance	“About the disclaimer again. Just say this involves questions about your medical history. If you want to, before answering, maybe talk with a relative or … If you have any questions, have someone close by you can ask.” (Incompletion; Interviewee 3)
Health literacy	•Add more literacy aids	“Just having like a little diagram of a woman’s body or, you know, a body with female organs and you have the uterus, ovaries and show like this is where the cervix is.” (Accuracy; Interviewee 52)
Language availability	• Make Spanish option more obvious; add option to toggle between English/Spanish	“Yeah [a toggle option would help]. Probably in the same questions with a little Spanish writing instead of going… taking full Spanish or full English–-have them both in the same website. And you can read in English if you prefer a language, or Spanish in the background.” (Time/Accuracy; Interviewee 38)
Interruptions or time barriers, length or detail of the tool	• Add clear notification about time required to complete and to find a quiet, uninterrupted place	“Maybe prepare me for, say the questions may be somewhat complex and you need to be…uninterrupted so you can focus and fully understand to provide accurate…You know, more proper answers.” (Accuracy; Interviewee 47)
Basic application functions	• Adding save and return function, back button, progress bar, overt symbols for hyperlinks, and automated reminders to return	“I think just to be able to go back instead of restarting….And so if I could just go back to the questions that needed to be changed, that would have been a little bit more helpful.“ (Incompletion; Interviewee 7)

The ability to gather family history from family members was also frequently cited both as a barrier to, and facilitator of, patient-facing family history assessment. Some individuals noted that their ability to complete the assessment hinged on the fact that they were able to obtain family history from their family members. However, most individuals identified this as a barrier, citing inability to obtain some part of family history due to death, estrangement, and/or geographical or language barriers between the participant and their family:

“You know, and…my mom [died]…so there’s a lot of things that my mom didn’t share with me about, you know, my grandmother’s history. And…my mom grew up without a dad, so I don’t know a lot about…my grandpa.” (Accuracy, Interviewee 48)

Family dynamics – including intrafamilial communication norms and cultural beliefs – sometimes influenced a participant’s ability to access family history information. Participants in the incompletion and time cohorts also frequently noted their desire to provide a higher level of accuracy than their knowledge would permit:

“I’m like, I am botching this up and I’m not giving accurate information. I’m just guessing at this point…So I think that’s probably when I was like this is worthless. Let me give this up.” (Incompletion, Interviewee 15)

By contrast, many individuals in the accuracy cohort indicated some level of comfort with providing best guesses, though many stated they would have sought accurate information in advance had they known the level of detail needed (e.g., specific type of cancer, age of diagnosis).

Participants suggested that having “I don’t know” or “not sure” options would alleviate distress around lack of access and reduce inaccuracy and guesswork. Some participants also suggested or endorsed starting with the module on limited family history knowledge for all individuals and using these responses to skip questions regarding relatives about whom they had no information (Table 2).

#### Electronic tool accessibility

Most individuals felt that the CHARM study family history assessment was predominantly straightforward and easy to understand, and that the tool’s use of lay and medical terms was helpful. However, terminology was noted as a barrier for many, especially among those in the accuracy cohort, who sometimes reported misunderstanding certain cancer types (i.e., reproductive cancers; whether metastatic cancer sites should be reported). Several participants suggested or endorsed addition of further literacy aids and specifying that questions were asking where cancer started and not where cancer spread (Table 2).

Language was also often noted as a barrier. For several participants, English was not their primary language or was not the primary language of their family. A few participants identified Spanish as their native language during the interview and had not realized they could have used a Spanish-language version. These participants suggested greater user interface visibility of the Spanish option and adding the option to toggle individual questions between Spanish and English (Table 2). One participant noted that if the Spanish option was more visible, they would have felt more respected by the study.

Most participants said the assessment took an expected or shorter-than-expected amount of time to complete. However, some participants in the incompletion and time cohorts shared that interruptions, lack of time, and/or the length or detail of the tool sometimes caused them to stop or take more time on the tool. Some suggested providing explicit and clear notifications of the need to find a quiet place to complete the assessment and the amount of time the tool would take (Table 2).

#### Electronic application functions

Most participants noted that the assessment was easy to use and navigation was smooth and intuitive. Participants used a wide variety of devices, and most indicated that the design and layout functioned well on their device:

“It loaded fine and it wasn’t squished or anything. It was great…. it was just very clearly laid out. And I like when there’s not a million questions on every screen. So that was well done.” (Incompletion; Interviewee 4)

Participants noted missing functions that would have helped them overcome barriers (Table 2). The most frequently desired functions were allowing participants to save their place and return without starting over and a back button so they could revisit and alter previous answers. A few interviewees also suggested adding a progress bar approximating how many questions remained. Finally, participants with incomplete attempts suggested or endorsed automated messages reminding them to return.

### Acceptability of study tool compared to clinician-collected family history

We assessed whether interviewees – who all experienced barriers to assessment – viewed the study tool as an acceptable alternative to clinician-collected family history by asking about experiences with family history collection in clinical care and preferences between the study tool versus a clinician.

#### Experiences with the electronic tool vs. clinician-collected family history

When participants compared experiences using a patient-facing assessment to experiences of clinician-collected family history, two major themes emerged: experiences of 1) respect and/or relationship and 2) family history collection approaches.

Regarding experiences of respect and relationships, some participants indicated ease of speaking with clinicians due to trust in the medical profession, while others indicated that trust depends on the individual clinician, with some making them feel heard and others being dismissive. Some participants highlighted past experiences of feeling disrespected by clinicians, such as being ridiculed for being concerned about family history or being told family eating habits caused cancer in their relatives because of the patient’s weight. One participant compared their experiences within clinical care at one study site to those in a care system designed for Native Americans, where they felt their family cancer history concerns were more validated and respected.

“But when I had the experience of just requesting and getting denied for the mammogram through my PCP [at primary study site], then I kind of felt unheard or like my concerns at this point don't matter...I'm just like [at the Native American health service organization, they are] not really like just giving everybody what they want, but [are] listening to them and doing it by ... a case by case [basis].” (Accuracy, Interviewee 54)

This participant also compared their experience with the study tool to their experience within the care system designed for Native Americans, indicating that the tool felt similarly respectful because it led to appropriate follow-up.

Most participants recalled discussing family history with a clinician at some point. While some participants noted a clinician’s ability to probe and explain concepts or have a ‘back-and-forth’ with the patient, clinicians were more commonly described as inattentive to detail or as treating the family history as unimportant:

“Like it doesn’t have an impact. Like it hasn’t…They haven’t said, oh okay. Well, we might want to talk about, you know…[chuckles]…keeping that on our radar or anything like that. It’s just like, okay, I’ll make a note.” (Accuracy, Interviewee 52)

By contrast, the assessment used in CHARM was typically described as more comprehensive and as allowing participants to spend more time carefully answering questions than they could in a clinical visit. A few participants noted patient-facing assessments offer privacy for topics that were uncomfortable for them (e.g., deaths, gynecologic cancers).

#### Preferences between the electronic tool and clinician-collected family history

Fifty-three percent (*N* = 28) of interviewees stated a preference for the CHARM tool over clinician-collected family history, while 15% (*N* = 8) stated a clinician preference, 13% (*N* = 7) stated that they did not have a preference, and 23% (*N* = 12) did not clearly commit. Preference distributions were similar across cohorts, with the highest preference for the clinician (26%, 5/19 respondents) in the time cohort, and among those in the incompletion cohort who had not completed the assessment (20%, 2/10).

A few participants who indicated a clinician preference noted that clinicians could interactively explain concepts, reducing their mental burden and increasing understanding. Others noted the ability of clinicians to tailor questions to their health, concern that online forms would not impact their care, personal comfort with face-to-face interactions, and/or a dislike of reading.

Common reasons for study tool preference included the opportunity to gather more accurate family history from relatives, the convenience and privacy offered, and the tool’s self-paced nature. Interviewees reported not feeling rushed when using the tool and/or being able to provide more detail, compared to limited time during a doctor’s appointment.

“There was more detail on the [tool]. And I could go at my own pace. And you know, a doctor’s appointment is rushed. So usually I just pinpoint like, you know grandparents, siblings, parents…because I know…there’s not a lot of time for the appointment. It feels so rushed. Yeah, time is limited.” (Accuracy, Interviewee 47)

Those who did not indicate a clear preference or stated they had no preference provided several reasons, including that preferences may depend on circumstances (e.g., patient-clinician relationship) or that both approaches had benefits and drawbacks. Benefits and drawbacks provided were similar to those cited by those with explicit preferences.

## Discussion

Given ever-increasing time pressure on primary care clinicians [40, 41], it is unsurprising that cancer family history information is rarely collected in sufficient detail to facilitate risk recognition [18–20, 42]. Electronic family health history tools can reduce burden on primary care clinicians, but it is critical to develop digital tools in ways that advance health equity [24, 43]. We developed a literacy-adapted patient-facing family history assessment designed to bridge care gaps for hereditary cancer risk recognition and referral that disproportionately impact patients from marginalized groups and utilized that tool as part of a multimodal intervention designed to increase access to hereditary cancer genetic testing in a population enriched for individuals from medically underserved populations [31, 32].

Although we specifically recruited interviewees who faced barriers to study tool use, a majority preferred the study tool to a clinician, frequently noting time pressure in clinical appointments. Several participants also recalled negative past experiences during clinician-collected family history. Given the CHARM study’s intentional recruitment of individuals from medically underserved populations, these participant experiences may reflect experiences of marginalization within large health systems that could contribute to observed disparities. Using inclusively designed patient-facing digital tools to improve care could help reduce experiences of medical marginalization in underserved communities.

Participant-reported barriers to the study tool and their proffered solutions provide easy-to-implement, broadly applicable guidelines for future design of inclusive patient-facing digital family history assessments and other digital health tools. For instance, participants described needing clear indications to gather family history prior to starting, an approach that has been employed by other tools [44], and provided solutions (Table 2) for participants without access to parts of their family history. Participants appreciated literacy-adaptation and identified words and concepts (e.g., reproductive cancers, metastatic cancers) that could benefit from additional literacy aids, including definitions and diagrams. Bilingual participants highlighted the need for prominent interface elements providing language options and suggested that tools offer the ability to toggle questions between languages. Participants also highlighted the importance of employing mobile-first and responsive design for use on a variety of devices, and the need for features that facilitate editing of responses.

Overall, 63% of interviewees identified as a race or ethnicity other than non-Hispanic White (with no provided information for 7% of participants), an improvement on recruiting patients from marginalized racial/ethnic backgrounds compared to many genetics studies [45]. Women were overrepresented in our interview cohort, reflecting their overrepresentation in the study overall. Because interviews were conducted only with participants interacting with the English-language version of the tool, our ability to recruit interviewees from the safety-net study site was limited [31]. As a result, most interviewees were insured, and 69% held a bachelor’s degree or higher, significantly higher than the nationally reported proportion of adults over age 25 with a bachelor’s degree (36%) in 2019 [46]. Accordingly, we also ascertained a low number of bilingual Spanish speakers and no one who viewed the Spanish-language version of the tool. While most solutions identified could be extended to the Spanish-language version, it is possible there are additional barriers for Spanish-language tools and for participants with lower educational attainment. While we provided a literacy- and culturally-adapted Spanish translation, we could not account for all languages and some interviewees spoke languages other than English or Spanish as their primary language. Ideally, culturally-adapted translations by certified translators for languages prevalent in the community should be created [47]. However, to account for the diversity of languages, future family history tools could leverage embedded cloud translation services to offer translation options for additional languages with appropriate human oversight to ensure accuracy of those translations.

## Conclusions

Electronic, patient-facing family history assessments are an acceptable, useful, and often preferred alternative to clinician-gathered family history and risk assessment in health care settings serving diverse populations, and participants identified easy-to-implement solutions for most barriers identified. Participants appreciated the use of lay language, literacy aids, and responsive design. They highlighted further opportunities for inclusive design, including clearer instructions, more literacy aids, skip logic changes to address lack of family history access, and application features that facilitate revision of responses.

## Supplementary Information

**Additional file 1.** Interview guide questions by topic. Contains all questions in semi-structured interview guide utilized by interviewer (KFM).

**Additional file 2.** Codebook definitions of all qualitative codes used. Contains the codebook codes and code definitions utilized by coders during analysis.

**Additional file 3.** Interviewee characteristics on per-cohort basis. Contains the information provided in Table 1, subdivided into the four cohorts described in this study.

## Data Availability

Data generated or analysed during this study are included in this published article and its supplementary information files or are available in de-identified form from the corresponding author upon reasonable request.

## Abbreviations

CHARM: Cancer Health Assessments Reaching Many
USPSTF: United States Preventive Services Task Force
HBOC: Hereditary breast and ovarian cancer syndrome
KPNW: Kaiser Permanente Northwest
DH: Denver Health
CSER: Clinical Sequencing Evidence-Generating Research
IRB: Institutional Review Board
LS: Lynch syndrome
